# A Review of Potential Harmful Interactions between Anticoagulant/Antiplatelet Agents and Chinese Herbal Medicines

**DOI:** 10.1371/journal.pone.0064255

**Published:** 2013-05-09

**Authors:** Hsin-Hui Tsai, Hsiang-Wen Lin, Ying-Hung Lu, Yi-Ling Chen, Gail B. Mahady

**Affiliations:** 1 School of Pharmacy and Graduate Institute, College of Pharmacy, China Medical University, Taichung, Taiwan; 2 Department of Pharmacy, China Medical University Hospital, Taichung, Taiwan; 3 Department of Pharmacy Administration, College of Pharmacy, University of Illinois at Chicago, Chicago, Illinois, United States of America; 4 Department of Pharmacy Practice, College of Pharmacy, PAHO/WHO Collaborating Centre for Traditional Medicine, University of Illinois at Chicago, Chicago, Illinois, United States of America; Royal College of Surgeons, Ireland

## Abstract

**Background:**

The risks attributed to drug-herb interactions, even when known, are often ignored or underestimated, especially for those involving anti-clotting drugs and Chinese medicines. The aim of this study was to structurally search and evaluate the existing evidence-based data associated with potential drug interactions between anticoagulant/antiplatelet drugs and Chinese herbal medicines (CHMs) and evaluate the documented mechanisms, consequences, and/or severity of interactions.

**Methodology and Findings:**

Information related to anticoagulant/antiplatelet drug-CHM interactions was retrieved from eight interaction-based textbooks, four web resources and available primary biomedical literature. The primary literature searches were conducted in English and/or Chinese from January 2000 through December 2011 using the secondary databases (e.g., PubMed, Airiti Library, China Journal full-text database). The search terms included the corresponding medical subject headings and key words. Herbs or natural products not used as a single entity CHM or in Chinese Medicinal Prescriptions were excluded from further review. The corresponding mechanisms and severity ratings of interactions were retrieved using *MicroMedex®*, *Lexicomp*® and *Natural Medicines Comprehensive Database*®. Finally, we found 90 single entity CHMs contributed to 306 documented drug-CHM interactions. A total of 194 (63.4%) interactions were verified for its evidence describing possible mechanisms and severity. Of them, 155 interactions (79.9%) were attributable to pharmacodynamic interactions, and almost all were rated as moderate to severe interactions. The major consequences of these interactions were increased bleeding risks due to the additive anticoagulant or antiplatelet effects of the CHMs, specifically danshen, dong quai, ginger, ginkgo, licorice, and turmeric.

**Conclusions/Significance:**

Conventional anticoagulants and antiplatelet drugs were documented to have harmful interactions with some commonly used single entity CHMs. For those patients who are taking conventional anti-clotting medications with CHMs for cardiovascular or cerebrovascular diseases, the potential risks of increased bleeding due to drug-CHM interactions should not be ignored.

## Introduction

Ischemic heart disease and stroke are the two primary causes of death worldwide, accounting for 24% of all deaths reported in 2008 [1]. Anticoagulants and antiplatelet drugs are important standard therapies used to prevent clot formation in the treatment and prevention of cardiovascular and cerebrovascular diseases [2]. However, most anticoagulant and antiplatelet agents have drug interactions with a variety of other medications, foods, and dietary supplements [3], [4]. A systematic review published in 2008 reported that medications with anticoagulant or antiplatelet activity are most likely to have interactions with herbal medicines [5]. Specifically, those patients given anticoagulant agents are at a higher risk of suffering from potentially harmful drug interactions, compared to other cardiovascular drugs, when co-administered with herbal medicines [6].

The use of complementary and alternative medicine (CAM), including herbal medicines, is widespread and has increased worldwide over the last decade [7], [8]. A systematic review of CAM found that the prevalence of CAM use varied widely from 9% to 65% [9]. Nearly 40% of patients with cardiovascular disease or stroke had used CAM therapies concomitantly with their prescribed medications [10], [11]. Traditional Chinese Medicine (TCM) is one of the dominant forms of CAM for many Asian populations [7], [12], [13], including those who migrated to western countries. A substantial percentage of Asian patients were likely to use TCM in combination with the conventional medicines [14]–[17]. For instance, 13% of patients using anticoagulant or antiplatelet agents were also prescribed concentrated CHMs concomitantly in a Taiwanese population-based study [17].

To date, there is very limited documented information about potential prescription drug interactions with CHMs. Considerable proportions of clinical practitioners or patients underreport their use of natural health products, including CHMs [18]. The majority of physicians and professional trainees have limited training on herbal adverse events, toxicities, and drug interactions [19]. The lack of training and education may be associated with a reduced recognition of potential drug-herb interactions, specifically their occurrence and associated adverse events. While patients taking anticoagulant/antiplatelet drugs are more likely to be exposed to potential drug-herb interactions (i.e., increased risks of bleeding [20]), the aim of this study was to systematically review the available published evidence-based data and biomedical reports associated with drug interactions between anticoagulant/antiplatelet drugs and CHMs, and further to evaluate the documented mechanisms, consequences, and/or severity of interactions.

## Materials and Methods

### Evidence retrieval and literature search

The evidence regarding “harmful” interactions between anticoagulant/antiplatelet drugs and herbal medications used in TCM (in terms of Chinese herbal medicine [CHM]) was the main focus and extracted from eight interaction-based textbooks [21]–[28], four web resources [29]–[32], and available primary biomedical literature sources. In this review, we defined CHM as the same definition derived from the web site of National Center for Complementary and Alternative Medicine and originally cited from Chinese Materia Medica [33]. The search of primary articles was conducted in four English and three Chinese secondary databases, including MEDLINE, PubMed, EMBASE, Cochrane Library, Airiti Library, Index to Taiwan Periodical Literature System, and the China Journal full-text database. The search terms in these databases included the corresponding medical subject headings (MeSH terms) and key words as follows: ‘herb drug interactions’; ‘anticoagulants’ OR ‘antiplatelet drugs’ OR ‘warfarin’ OR ‘heparin’ OR ‘aspirin’ OR ‘clopidogrel’ OR ‘dipyridamole’ OR ‘ticlopidine’ AND ‘Traditional Chinese Medicine’ OR ‘Chinese medication’ OR ‘herbal medicine’ OR ‘phytotherapy’ AND ‘interaction.’ The searches were restricted to either the English or Chinese language during 2000 to 2011 (from January 2000 through December 2011). The articles were selected based on the title and abstract. The selected articles were retrieved independently by two authors (YLC, YHL), and then validated by the other two authors (HHT, HWL). Specifically, all retrieved literature were selected regardless of types of study, i.e., animal studies, clinical trials, observational studies, or review articles. Those in vitro studies or literature without interaction reports, or corresponding studies about pure compounds (but not for single entity CHMs), were excluded.

### Evidence extraction and review

Initially, any level of evidence-based interactions, as long as it was retrieved from the aforementioned books, web sites, or primary literature, between the anticoagulant/antiplatelet agents and any natural products or herbs (i.e., either being classified as CHMs or not) was first extracted and documented. However, animal-derived medications used in TCM were not included. Then, we used a standardized data abstraction checklist on *Excel®* spreadsheet to extract all relevant data, including common name, scientific name and/or binomial source, plant parts, dose, route of administration, drug name, consequence of interactions, and evidence resources, etc, if they were available. Data for all anticoagulant/antiplatelet agents was extracted regardless of belonging to a drug class or an individual drug. The anticoagulants were composed of heparin and warfarin while antiplatelet agents included aspirin, clopidogrel, dipyridamole and ticlopidine. All relevant data in the literature was extracted and compiled by two of the authors (YLC, YHL), and then validated by the other two authors (HHT, HWL). To cross-validate the retrieved and reviewed information, we conducted the focus group review and discussions. A focus group included five experienced pharmacists practicing in either Western medicine or Chinese medicine on a daily basis in Taiwanese hospitals. Any disagreements about classifications and information conflict were resolved by consensus. Specifically, all of the members in the focus group were trained in both Western and Chinese medicines in a School of Pharmacy and/or their master graduate programs. They all have more than 15 years of experience practicing in TCM pharmacy or clinical pharmacy services in hospitals that provide medical services with both Western and TCM medicines. None of these members were authors.

### Data management and analysis

Next, the following review and analysis particularly focused on those single entity CHMs commonly listed in Chinese Medicine books, including the Taiwan Herbal Pharmacopoeia (THP) [34], Chinese Medicinal Herbs Preparation [35], and Chinese Materia Medica [36]. Thus, those natural products or herbs that were not documented in the aforementioned books and/or any combination of Chinese Medicinal Prescriptions, which were listed in some ancient TCM books, were excluded for further analyses. Moreover, those single entity CHMs included for further review were verified by two experienced pharmacists who were members of the focus group.

To obtain proof about clinical significance of interactions, we identified and verified the severity ratings and possible mechanisms of each interaction using three well-known interactions databases (*MicroMedex®* [37], Lexi-Interact in *Lexicomp®* [38], or “Natural Product/Drug Interaction Checker” in *Natural Medicines Comprehensive Database® (NMCD®*) [39]). Because of the various definitions of severity ratings among these three databases, we compiled the rating schemes and categorized the severity of each interaction into six types in this review: “contraindicated,” “major,” “moderate,” “minor,” “no interaction,” and “no available information for the item.” The comparisons of severity rating definitions in different databases with this review were listed in Table 1. Accordingly, the mechanisms for interactions were also categorized into pharmacokinetics, pharmacodynamics, pharmacokinetics plus pharmacodynamics, and unknown based upon the records from these three databases. All of the related data was compiled and managed using the *Excel®* spreadsheets. Last, the descriptive analyses were performed to explore the frequency and proportion of the retrieved evidence associated with the interaction numbers, the corresponding types of retrieved mechanisms and severity ratings of interactions.

**Table 1:** The comparisons of severity rating definitions in different databases with current review.

	MicroMedex®	Lexicomp®	NMCD®	Current review
Contraindicated	Do not use combination; contraindicated.	-	-	Interactions with severity rated as “contraindicated” according to MicroMedex®.
Major	Combination may cause life-threatening damage and/or need medical intervention to prevent severe adverse effect.	Effects may result in death, hospitalization, permanent injury, or therapeutic failure.	Do not use combination; contraindicated; strongly discourage patients from using this combination; a serious adverse outcome could occur.	Interactions with severity rated as “major” in any of the three databases.
Moderate	Combination may cause worsen the patient’s condition and/or need to change the therapy.	Medical intervention needed to treat effects; effects do not meet criteria for Major.	Use cautiously or avoid combination; warn patients that a significant interaction or adverse outcome could occur.	Interactions rated as “moderate” and without “major” score in any of these databases.
Minor	Do not need to change the therapy.	Effects would be considered tolerable in most cases; no need for medical intervention.	Be aware that there is a chance of an interaction; advise patients to watch for warning signs of a potential interaction.	Interactions rated as “minor” and without “moderate” or “major” score in any of these databases.
No interaction	-	-	-	There was no documented interaction between the medication and the single entity CHM
No available documented information for the item	-	-	-	There was no available information about the single entity CHM in these databases.

NMCD: Natural Medicines Comprehensive Database.

## Result

### Literature search and evidence retrieval

**Figure 1:**
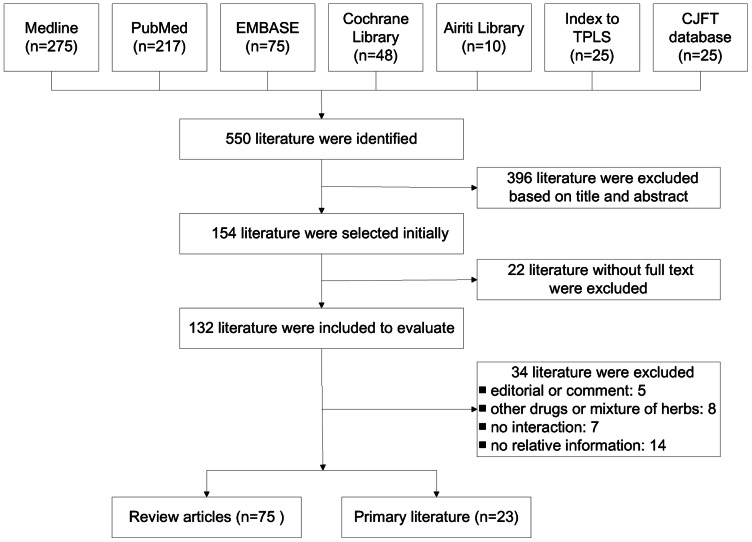
Flow chart of primary literature search. TPLS: Taiwan Periodical Literature System; CJFT: China Journals Full-text.

A total of 154 articles were selected from the initial 550 retrieved literature reports based on their relevance to the purpose of review. After thorough evaluation and analysis, 98 articles with full text (75 review articles [5], [6], [40]–[112], and 23 primary studies, i.e., 4 animal studies [113]–[116], 4 clinical trials [117]–[120], and 15 observational studies [20], [121]–[134]) were included for further evaluation (Figure 1). The summaries of retrieved primary literature are listed in Table 2, while the summaries of retrieved review articles are listed in Appendix S1. The retrieved data missing information concerning the common name, scientific name, plant source, plant parts, and dose for the natural products/herbs or CHM were excluded from Table 2 and Appendix S1, but may be accessed upon specific request to the authors.

**Table 2:** Summary of the included primary literature and the retrieved relevant information about natural products or herbs (including CHMs).

Clinical trials					
Reference	Natural products/ herbs (including CHMs)	Dose schedule of Natural products/herbs (including CHMs)	Medication	Study design	Observed/measured outcomes
Abdul 2010 [117]	Commercial products of echinacea (oral)	14 days	Warfarin (oral)	Open-label, randomized, three-treatment, cross- over study	INR, platelet activity, Warfarin concentration
Jiang 2006 [118]	Commercial products of St John’s wort, Asian ginseng, ginkgo, or ginger (oral)	7 or 14 days	Warfarin (oral)	Two randomized, open-label, controlled, crossover studies	INR, Warfarin concentration
Jiang 2004 [119	Commercial products of St John's wort, ginseng (oral)	7 days or 14 days	Warfarin (oral)	Open-label, three-way crossover randomized study	Platelet aggregation, INR, Warfarin protein binding, Warfarin concentration
Yuan 2004 [120]	Root of American ginseng (oral)	3 weeks	Warfarin (oral)	Randomized, double-blind, placebo-controlled trial.	INR, Warfarin concentration

Animal studies					
Reference	Natural products/ herbs (including CHMs)	Preparation of Natural products/herbs (including CHMs)	Medication	Animal model	Observed/measured outcomes
Kuo 2006 [113]	Dried roots of danshen	Ethyl acetate extracts	7-Hydroxywarfarin	Mice	The effect on CYP
Li 2010 [114]	Dried fruits of danggui and honghua	Aqueous extracts	Clopidogrel	Rat	Bleeding time and hematology parameters
Makino 2002 [115]	Commercial product of Kangen-Karyu	Aqueous extracts	Warfarin	Rat	Plasma concentration, prothrombin time, bleeding time
Wu 2010 [116]	Commercial product of danshen	Ethyl acetate extracts	Warfarin	Rat	Pharmacokinetics parameters

Observational studies					
Reference	Natural products/herbs (including CHMs)	Medication	Study design	Population (number of participants)	Observed/measured outcomes
Blalock 2009 [121]	Herbs and natural products	Conventional medication	Population-based epidemiological study	Data from Johnston County Osteoarthritis Project (n = 2523)	Prevalence of exposure to potential interactions
Bush 2007 [122]	Herbal and dietary substances	General	Survey	Outpatients from clinics (n = 804)	The rate of potential and observed adverse herb-drug interactions
Canter 2004 [123]	Herbal and nutritional supplements	General	Self-completed survey	Elderly outpatients (n = 271)	Associated risks of negative interactions with prescription drugs
Chan 2011 [124]	Ginkgo biloba	Antiplatelet or anticoagulant agents	Retrospective population based study	Combination users of ginkgo and antiplatelet or anticoagulant agents (n = 3579 or 15397)	The prevalence and frequency of co-prescription, the relative risk of hemorrhage associated with combination
Chan 2011 [125]	Herbs	Warfarin	Survey	Patients with nonvalvular atrial fibrillation (n = 250)	Effect of concomitant herbal intake on anticoagulation control
Charrois 2007 [126]	Natural health products	General	Survey	Community pharmacists (n = 132)	Community pharmacists' knowledge of interactions
Clauson 2008 [127]	St. John's wort	General	Evaluation of label	-	Identification of labeling of 8 clinically relevant safety issues
Dergal 2002 [128]	Herbal medicines	Prescription and over- the-counter drugs	Survey	Older adults (≥65 years) attending a memory clinic (n = 195)	The frequency of potential interactions between herbal medicines and conventional drug therapies.
Goldman 2008 [129]	Natural health products	General	Survey	Parents and patients 0 to 18 years at a large pediatric emergency department (n = 1804)	The frequency of concurrent use and potential interactions
Rogers 2001 [130]	Herbs	General	Survey	Emergency department patients with heart disease, diabetes, psychiatric disorders, and/or hypertension (n = 944)	Prevalence and occurrence of sever herb-drug interactions.
Saw 2006 [20]	Herbs	Antiplatelet/anti coagulant drugs	Cross-sectional survey	Patients from cardiology, neurology, infectious and nephrology wards (n = 250)	Assessing the use of herbal medicines in medical patients that may interact with antiplatelet or anticoagulant therapy
Segal 2006 [131]	Matricaria chamomilla	Warfarin	Case report	70-year-old woman	-
Shalansky 2007 [132]	Complementary and alternative medicine	Warfarin	Prospective, longitudinal study	Adults prescribed warfarin for an expected duration of at least 4 months (n = 171)	Risk factors for bleeding events and supratherapeutic INR
Su 2010 [133]	Shengmai-yin	Warfarin	Case report	A 71-year-old man	-
Lai 2010 [134]	Chinese herbal medicines	Warfarin	Case-crossover study	Warfarin users from database (n = 4211)	Causal inference of potential interactions

CYP: cytochrome P450; VKORC1: vitamin K epoxide reductase complex subunit 1; INR: international normalized ratio.

**Figure 2:**
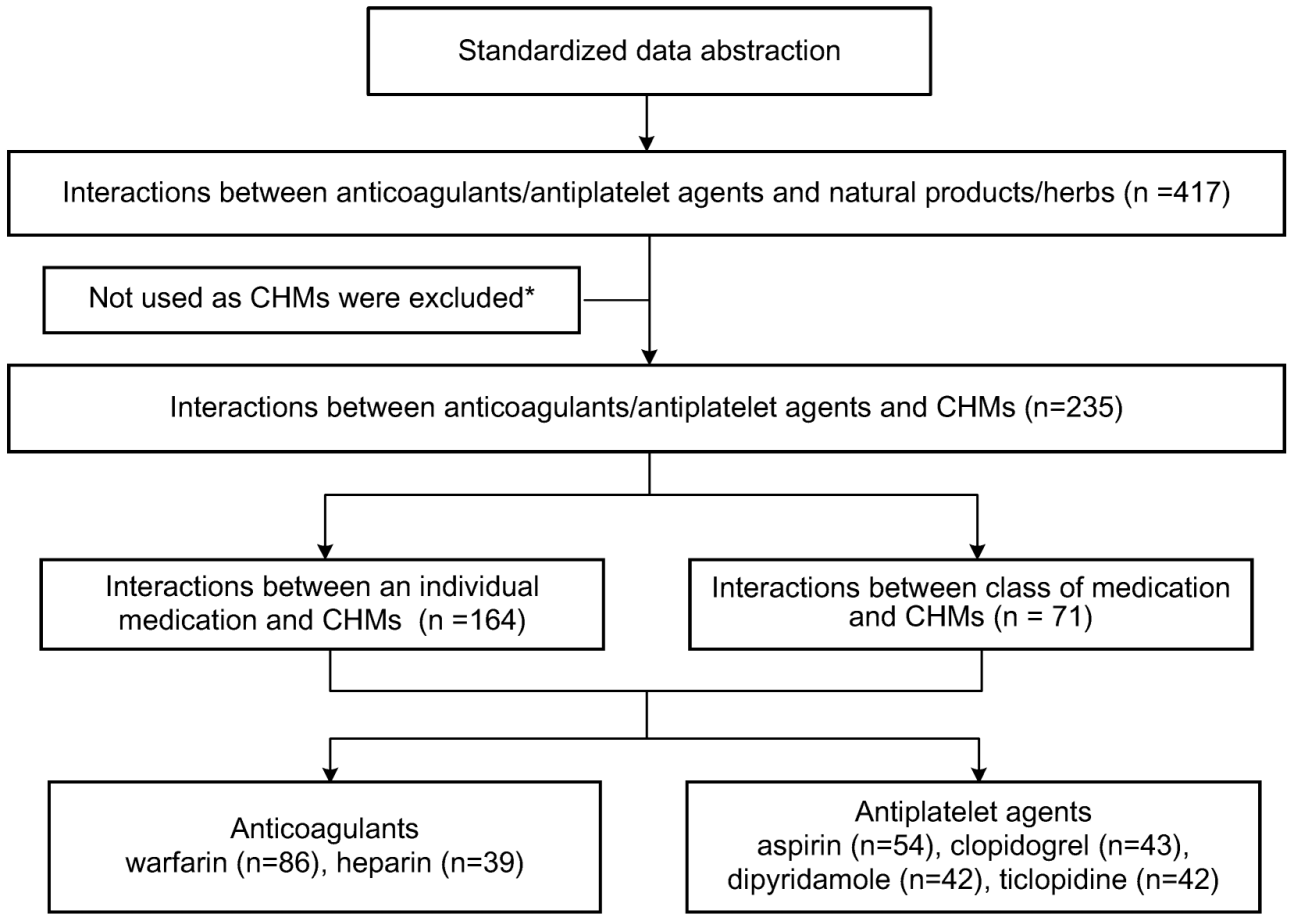
Retrieved findings of interactions between anticoagulant/antiplatelet agents and single Chinese herbal medicines. * Natural products or herbs which were not identified in the Taiwan Herbal Pharmacopoeia, Chinese Medicinal Herbs Preparation, or Chinese Materia Medica were excluded. CHMs: Chinese herbal medicines.

Initially, there were 417 interactions in total obtained from the aforementioned evidence-based resources. Of them, 235 interactions between anticoagulant/antiplatelet agents and specific single CHMs were extracted for further evaluation. After expanding the interactions to individual medications from the different drug classes, a total of 306 interactions between distinct anticoagulant/antiplatelet agents and single entity CHMs were identified. Warfarin and aspirin accounted for the majority of documented, evidenced-based interactions with single entity CHMs (Figure 2). Overall, 90 distinct single entity CHMs contributed to the 306 documented interactions.

### Severity rating and mechanisms upon availability in three databases

**Figure 3:**
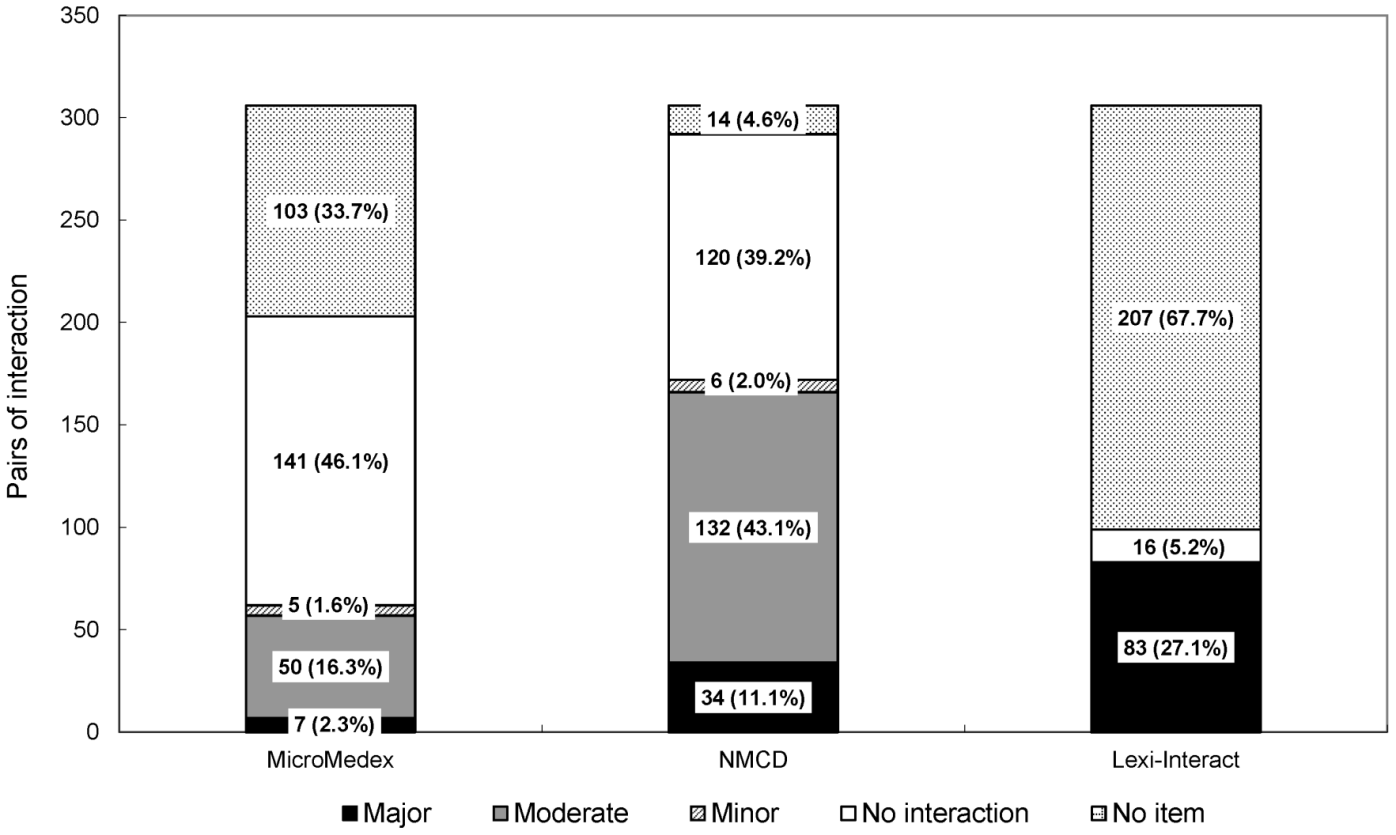
Documented severity ratings of interactions between distinct anticoagulant/antiplatelet agents and single Chinese herbal medicines. The total interactions between distinct anticoagulant/antiplatelet agents and single CHMs were 306. The classification of “No interaction” meant that there is no interaction between the medication and the single CHMs, while “No item” meant that there was no available information about the single CHMs in the database. NMCD: Natural Medicines Comprehensive Database.

Of the 306 interactions between distinct anticoagulant/antiplatelet agents and single entity CHMs, 203 (66.3%), 292 (95.4%), and 99 (32.3%) drug-CHM interactions were retrieved with some relevant information about interactions from *MicroMedex®*, *NMCD®*, and *Lexicomp®*, respectively. However, only 62 (20.2%), 172 (56.2%), and 83 (27.1%) of these interactions in *MicroMedex®*, *NMCD®*, and *Lexicomp®*, respectively, contained identifiable severity ratings and interaction mechanisms (Figure 3). Overall, a total of 194 (63.4%) interactions were found to have severity ratings and mechanisms in at least one of these databases. While 50 interactions with information in *MicroMedex®* (80.6%) were categorized as the moderate interactions, 132 interactions documented in *NMCD®* (76.7%) were classified as moderate. However, all of the 83 documented interactions in *Lexicomp®* were found to have major drug-CHM interactions (Figure 3). Consequently, of all 194 interactions being identified with the severity rating in any of the three databases, only 29 (15.0%) were found in all of the three databases, 65 (33.5%) could be identified in two databases, while more than half (100, 51.5%) were documented in only one database.

Of those identifiable mechanisms for interactions, the majority (155, 79.9%) were attributable to pharmacodynamic-related mechanisms. 8.3% were attributed to pharmacokinetic-related mechanisms and 11.3% were the results of both pharmacodynamic and pharmacokinetic mechanisms. Most of the pharmacodynamic-related interactions were due to the additive anticoagulant/antiplatelet effect of the single entity CHM. For example, some CHMs, i.e., clove (*Eugenia caryophyllata*), cat's claw (*Uncaria tomentosa*), and ginger (*Zingiber officinale*), were reported to inhibit cyclooxygenase activity, platelet aggregation, and thromboxane synthetase activity, respectively [37], [39]. Most of these potential interactions have not been documented in clinical practice. In contrast, a few documented single entity CHMs with pharmacodynamic-related interactions causing decreased anticoagulant effects were reported in the literature. For example, alfalfa (*Medicago sativa*) and green tea (*Camellia sinensis*) may antagonize the anticoagulant effects of warfarin due to the presence of vitamin K in these products [37], [39]. As for the documented pharmacokinetic-related interactions, the majority were due to the inhibition of warfarin or clopidogrel metabolism *via* the Cytochrome P450 (CYP) pathway, including CYP 1A2, 3A4, and 2C9. For example, dandelion (*Taraxacum officinale*), bitter orange (*Citrus aurantium*), and milk thistle (*Silybum marianum*) were reported to inhibit CYP1A2, 3A4, and 2C9, respectively. Therefore, these herbal products may inhibit warfarin or clopidogrel catabolism, and result in an increase in their anticoagulation/antiplatelet activities [39]. Similarly, ginkgo (*Ginkgo biloba*), red clover (*Trifolium pratense*), and Siberian ginseng (*Eleutherococcus senticosus*) were documented to inhibit all of these three CYP enzymes, thus similar effects on the anticoagulation action of warfarin or clopidogrel may be expected [37], [39].

### Consequences of drug–CHM Interactions

Of the 90 interactions that involve a single entity CHM, the majority would result in an increased risk of bleeding when used in combination with anticoagulants or antiplatelet agents (Table 3). For instance, commonly used CHMs including danshen (*Salvia miltiorrhiza*), dong quai (*Angelica sinensis*), ginger (*Zingiber officinale*), and licorice (*Glycyrrhizae uralensis*) were documented to have major interactions with anticoagulants or antiplatelet drugs. The combinations causing major interactions may cause life-threatening damage and/or severe adverse events. Thus, we strongly discourage patients and clinicians from using these combinations [37]–[39]. Specifically, celery, a functional food and classified as a CHM in the Chinese Materia Medica, is reported to increase bleeding in Philp's book and a few review articles and was then verified in the *Lexicomp®* record [26], [81], [82], [85], [94]. There were no published primary data that describe the rationale or potential mechanism of this interaction. Therefore, interpretation of the data related to celery should be cautious.

**Table 3:** Documented interactions between anticoagulant/antiplatelet drugs and Chinese herbal medicine, a which might increase the risks of bleeding.

Severity rating ^b^	CHM documented to increase bleeding risks of the six anticoagulant/antiplatelet drugs	CHM documented to increase bleeding risks of at least one of the six anticoagulant/antiplatelet drugs
Major	Celery, Chamomile, Danshen, Dong quai, Evening primrose, Fenugreek, Garlic, Ginger, Ginkgo, Horse chestnut, Licorice, Red clover, Reishi,^c^ Turmeric, Willow	Cat's claw
Moderate	Andrographis, Bogbean, Cayenne, Clove, Flaxseed, Kudzu, Onion, Saw palmetto	Aloe vera, Asafetida, Bitter orange, Blackcurrant seed, Burdock, Cassia, Cinchona, Coltsfoot, Da huang, Eucalyptus, Lycium, Milk thistle, Nutmeg, Peony, Royal jelly, Safflower, Soybean, Valerian, Yarrow
No available information for the item	Artichoke, Astragalus, Dandelion,^d^ Glehnia, Kelp, Lavender, Meadowsweet,^e^ Red yeast rice,^f^ Skullcap	American ginseng, Angelica,^f^ Artemesia, Black cohosh,^f^ Chrysanthemum flower, Corydalis yanhuso, Dang shen, Erigeron, Geum japonicum, Hawthorn,^e^ Horseradish, Jamaica quassia, Japanese brome, Lovage, Magnolia bark, Motherwort, Plantain, Pubescent angelica, Rue, Shiitake, Sweet melilot, Tamarind,^e^ Wood ear mushrooms

a Chinese herbal medicine were identified according to Taiwan Herbal Pharmacopoeia,[34] Chinese Medicinal Herbs Preparation,[35] or Chinese Materia Medica.[36]. ^b^ The severity rating was categorized according to *MicroMedex®*, *Lexicomp®* and *Natural Medicines Comprehensive Database®.* Interactions with severity rated as major in any of the three databases were included in the “major” type, while interactions rated as moderate and without major score in any database were included in the “moderate” type. “No available documented information for the item” meant that there was no available information about the single CHM in these databases. ^c^ Interaction with heparin is lack of information about severity rating. ^d^ Interaction with clopidogrel or warfarin is moderate. ^e^ Interaction with aspirin is moderate. ^f^ Interaction with warfarin is moderate.

Furthermore, a few reports showed that single entity CHMs may decrease the effectiveness of anticoagulant/antiplatelet agents. Agrimony (*Agrimonia eupatoria*), myrrh (*Commiphora myrrha*), and St. John's wort (*Hypericum perforatum*) were documented to result in a decreased effectiveness of warfarin [21], [22], [24], [25], [72], [135], while pepper (*Piper nigrum*) and cannabis (*Cannabis sativa*) were reported to reduce the antiplatelet efficacy of aspirin [28]. Nevertheless, we also noted the conflicting consequences of interactions reported in the different evidence resources. Some evidence suggested that alfalfa, Asian ginseng (*Panax ginseng*), green tea, and Siberian ginseng may reduce the anticoagulant effects of anticoagulant/antiplatelet agents [5], [37], [73], [74], while some published data suggested that these single entity CHMs might increase the risk of bleeding episodes if they were used concurrently with anticoagulants and antiplatelet drugs [38], [39], [73].

## Discussion

In this review, the potential interactions between anticoagulant/antiplatelet drugs and single entity CHMs were analyzed and evaluated based on retrievable and published evidence. We found 90 commonly used single entity CHMs (such as danshen, dong quai, ginger, and licorice) were involved in 306 evidence-based drug interactions with anticoagulant and/or antiplatelet drugs. Warfarin and aspirin were the two drugs reported to have the largest numbers of documented interactions with the greatest number of single entity CHMs. Most of these interactions were moderate to severe and attributable to pharmacodynamic mechanisms. The majority of these interactions were found to increase the risks of bleeding.

In the United States and Singapore, 10–20% of patients used herbal therapies or CHMs when using prescribed anticoagulant or antiplatelet medications for cardiovascular diseases or stroke [10], [11]. A survey also showed that one in five patients in Malaysia who took anticoagulant or antiplatelet drugs, also concurrently used herbal therapies [20]. While the use of TCM is prevalent among Asian populations [12], [13], there is also an increasing trend of using TCM in Western countries. Thus, it is necessary that clinicians be aware of these drug-CHMs interactions in order to effectively manage those patients who are using anticoagulant/antiplatelet drugs with the listed CHMs.

In this review, the majority of documented interactions were rated as “major or severe” in scale. However, the actual consequences of concurrent use might be changed depending on the used medications, dose levels, binomial source, plant parts, and/or route of administration of CHMs, in addition to the patients' characteristics (such as genetic differences and dietary habits), their use patterns, and combinations of single CHMs and/or Chinese Medicinal Prescriptions (based on TCM theory). As for the safety and effectiveness of using anticoagulants and antiplatelets, health care providers need to be more judicious in counseling their patients regarding the possibility of interactions between these drugs and herbal remedies or CHMs. If concurrent use is unavoidable, then health care providers should closely monitor patients for potential adverse events associated with these possible interactions (e.g., increased international normalized ratio, bleeding).

In fact, the interactions between herbal remedies or CHMs and drugs are often more complex due to the multiple ingredients found in all single entity CHMs [87], [94] and their binomial source, plant parts, dose, quality, preparation and/or route of administration [54]. Again, the likelihood of drug-CHM interactions may be different when using combinations of several single entity CHMs or those Chinese Medicinal Prescriptions. In particular, Chinese Medicinal Prescriptions (combined TCM medicines based on the theory of TCM) are sometimes the first line of therapy in Asian countries [136], [137]. For instance, Jia Wei Hiaxo Yao San is commonly used for anxiety, irritability, and depression, and is one of the most frequently used Chinese Medicinal Prescriptions in Taiwan. This well known ancient Chinese herbal formula is generally used for liver Qi stagnation but contains at least ten distinct entity of CHMs [137], including dong quai, ginger, and licorice, which are documented to have major interactions with anticoagulant or antiplatelet drugs [37]–[39]. Therefore, those patients using Jia Wei Hiaxo Yao San might encounter higher bleeding risks due to the occurrence of significant major interactions. In this case, health care professionals could more aggressively take appropriate actions to prevent them from causing serious life threatening adverse events in their patients if they were aware of such potential risks.

In this review, we also found that the major documented mechanisms of interactions between anticoagulant/antiplatelet drugs and CHMs were attributable to their pharmacodynamic interactions, especially due to additive anticoagulant or antiplatelet effects. Many of the commonly used CHMs such as clove, danshen, ginger, and licorice are documented to have intrinsic anticoagulant or antiplatelet properties [85], [138]. However, these blood-activating or stasis-resolving CHMs (e.g., danshen, and dong quai) are widely used in TCM for the treatment of cardiovascular or cerebrovascular diseases. Although some clinical studies have been conducted to investigate the effects of these CHMs on stroke or coronary diseases [139], [140], further large scale, rigorous clinical trials are needed to confirm the benefits and risks of using these CHMs. With limited robust evidence and clinical trials supporting the use of CHMs, it is crucial for physicians and pharmacy practitioners to educate their patients on why they need to disclose their concurrent use of conventional medications and CAM, including CHMs, especially for those patients who are taking anticoagulant or antiplatelet drugs.

Currently, there is no single comprehensive database to identify the facts of drug-CHM interactions, levels of evidence sufficiency, corresponding mechanism, and/or clinical significance. Instead, we utilized three commonly used natural products or drug interaction-related databases to identify and verify the information needed and retrieved this information regarding their mechanism and severity rating. The results of this review show that more than half of documented drug interactions retrieved from books, web sites, or primary literature could be identified in *NMCD®*, and less of them could be retrieved in *MicroMedex® or Lexicomp®*. This finding is consistent with the results of a previous study which was conducted in 2008 to evaluate the commonly used dietary supplement databases [141]. Of these databases, *NMCD®* had been recognized as the most appropriate database used to answer questions (including drug interactions) about dietary supplements [141]. In addition, we also found that only a few interactions could be identified in all of the tertiary literature (*MicroMedex®*, Drug interaction facts) and there existed variations in the severity rating of interactions [142]. This finding is also in agreement with Abarca's evaluation, which concluded that only a few major drug-drug interactions were listed in the four available interaction compendium and with very little agreement among them [142]. Thus, further rigorous qualitative studies that include opinions from a variety of experts on CHMs and drug interactions are needed to provide more consistent and robust information about the levels of clinical significance and mechanisms and combinations to be avoided to prevent drug-CHM interactions from occurring.

Nevertheless, our review has a number of limitations. First, there exists discordance in the nomenclature of Chinese medicine and herbs, as well as in both of the English and Chinese resources that need to be addressed. In particular, some evidence was retrieved from the English resources showing that an inconsistency of homonyms, synonyms, or translation may exist. Furthermore, only a small portion of the literature listed the common names of the natural products or herbal remedies, and the majority of them lacked any information on the scientific names and/or binomial source. Therefore, we need to be cautious when interpreting the findings on the natural products, herbs and CHMs per se. Second, the majority of literature reports did not specify which parts of natural products/herbs and CHMs (e.g., roots, leaves, seeds, or flowers) were used, on what doses, or which routes of administration were taken. In fact, the active ingredients and doses of CHMs may vary by situation and person based on TCM theory. Usually, the kinds and parts of CHMs and their preparation procedures are chosen based upon the stages of patients' disease status or condition. Lastly, only those interactions with evidence-based data retrieved from *MicroMedex®*, *NMCD®*, or *Lexicomp®*, were further reviewed for the corresponding mechanisms and rating of severity in this study. Thus, the interpretations might be subjective to information obtained from the retrieved database(s), and validated by the reviewers and members in the focus group. However, we have tried our best to screen and highlight the interactions using the best available evidence and consistent approach, although not all reports or studies were published or relevant documented interactions analyzed.

## Conclusions

Conventional anticoagulants and antiplatelet drugs were documented to have harmful interactions with some commonly used single entity CHMs. Concurrent use of some herbal remedies may increase or reduce the pharmacologic effects of anticoagulant and antiplatelet drugs with moderate or severe consequences. For those patients who are taking conventional anti-clotting medications with CHMs for cardiovascular or cerebrovascular diseases, the potential risks of increased bleeding due to drug-CHM interactions should not be ignored. This review provides some guidance to health care professionals as to how to recognize the potential risks of interactions between anticoagulants/antiplatelets and single entity CHMs. Moreover, it is critical for all health care professionals to be able to initiate and effectively communicate and discuss the proper use of CHM-related products with conventional medications (i.e., anticoagulant or antiplatelet agents for cardiovascular or cerebrovascular diseases) because many patients may not voluntarily disclose their CHM-related use to their physicians.

## Supporting Information

Appendix S1 **Summary of review articles to retrieve relevant information about interactions between anticoagulants/antiplatelet drugs and natural products/herbs (including CMHs).**
(DOCX)

Appendix S2 **PRISMA checklist.**
(DOCX)
