# Supplementary material for: A Review of Potential Harmful Interactions between Anticoagulant/Antiplatelet Agents and Chinese Herbal Medicines
Source: PLoS One. 2013 May 9;8(5):e64255. doi: 10.1371/journal.pone.0064255 (PMC3650066; doi:10.1371/journal.pone.0064255)
Supplement: Appendix S1 — Summary of review articles to retrieve relevant information about interactions between anticoagulants/antiplatelet drugs and natural products/herbs (including CMHs). (DOCX) [file pone.0064255.s001.docx]

**Appendix 1: Summary of review articles to retrieve relevant information about interactions between anticoagulants/antiplatelet drugs and natural products/herbs (including CMHs)**

| **Reference** | **Review type** | **Natural products/ herbs (including CHMs)** | **Medications** | **Databases** | **Searching period** |
| --- | --- | --- | --- | --- | --- |
| Awang 2002 [40] | Narrative review | Herbs | Cardiovascular Drugs | No mention | No mention |
| Bone 2008 [41] | Narrative review | Ginkgo biloba | Antiplatelets or anticoagulants | No mention | No mention |
| Borrelli 2009 [42] | Narrative review | St John’s wort | General | No mention | No mention |
| Brazier 2003 [43] | Systematic review | Herbs | General | MEDLINE, Allied and Complementary Medicine Database, CINHAL, HealthSTAR, and EMBASE | Their inception to 2001 |
| Bressler 2005 [44] | Narrative review | Saw palmetto | Prescription Medications | No mention | No mention |
| Bressler 2005 [45] | Narrative review | Ginseng | Prescription Medications | No mention | No mention |
| Bressler 2005 [46] | Narrative review | St. John's wort | Prescription Medications | No mention | No mention |
| Bressler 2005 [47] | Narrative review | Ginkgo biloba | Prescription Medications | No mention | No mention |
| Butterweck 2008 [48] | Narrative review | Herbs | General | No mention | No mention |
| Chan 2010 [49] | Narrative review | Traditional Chinese medicine | Western therapeutics | No mention | No mention |
| Chavez 2006 [50] | Narrative review | Herbs | General | No mention | No mention |
| Choi 2011 [51] | Narrative review | Herbs | General | No mention | No mention |
| Cohen 2010 [52] | Narrative review | Herbal supplements | Cardiovascular drugs | No mention | No mention |
| Colalto 2010 [53] | Narrative review | Herbs | General | No mention | No mention |
| Coxeter 2004 [54] | Narrative review | Herbs | General | No mention | No mention |
| De Smet 2008 [55] | Narrative review | Natural products | General | PubMed | 2005-2008 |
| Di 2001 [56] | Narrative review | St John’s wort | General | No mention | No mention |
| Di 2008 [57] | Narrative review | St John’s wort | General | No mention | No mention |
| Ernst 2000 [58] | Narrative review | Herbs | General | No mention | No mention |
| Ernst 2004 [59] | Narrative review | Herbal medicinal products | General | No mention | No mention |
| Fugh-Berman 2000 [60] | Narrative review | Herbs | General | MEDLINE; EMBASE | MEDLINE 1966–98; EMBASE 1994–99 |
| Fugh-Berman 2001 [61] | Systematic review | Herbs | Conventional drugs | MEDLINE, (via PubMed), EMBASE, the Cochrane Library, CISCOM | Their inception to 2000 |
| Gardiner 2008 [62] | Narrative review | Herbal, dietary Supplement | Anticoagulants, cardiovascular medications, psychiatric medications, laxatives, diabetes medications, or medications for human immunodeficiency virus (HIV) infection | No mention | No mention |
| Greenblatt 2005 [63] | Narrative review | Natural substances | Warafrin | No mention | No mention |
| Greeson 2001 [64] | Narrative review | St John’s wort | General | No mention | No mention |
| Haller 2006 [65] | Narrative review | Herbal and dietary supplements | General | No mention | No mention |
| Holcomb 2009 [66] | Narrative review | Herbs | General | No mention | No mention |
| Hu 2005 [67] | Narrative review | Herbs | General | MEDLINE, Biological Abstracts, Cochrane Library, AMED, Biosis Previews and EMBASE | Their inception to 2005 |
| Huang 2004 [68] | Narrative review | Dietary supplements | General | No mention | No mention |
| Izzo 2005 [69] | Narrative review | Herbs | General | No mention | No mention |
| Izzo 2005 [6] | Systematic review | Herbal medicines | Cardiovascular drugs | MEDLINE | 1966-2003 |
| Izzo 2001 [70] | Systematic review | Herbal medicines | Prescribed drugs | MEDLINE (via PubMed), EMBASE, Cochrane Library and phytobase | Their inception to 2000 |
| Izzo 2009 [71] | Systematic review | Herbal medicines | Prescribed drugs | MEDLINE (via PubMed), EMBASE and Cochrane Library | Their inception to 2009 |
| Javed 2008 [72] | Narrative review | Herbal medicines and nutritional supplements | Anticoagulants | MEDLINE (PubMed), UKMi database | Their inception to 2006. |
| Ko 2004 [73] | Narrative review | Traditional Chinese medicines | General | MEDLINE | 1966 to 2003 |
| Kuhn 2002 [74] | Narrative review | Herbs | General | No mention | No mention |
| Madabushi 2006 [75] | Narrative review | St. John’s wort | General | MEDLINE and EMBASE | Their inception to 2005 |
| Markowitz 2001 [76] | Narrative review | St . J oh n’s wort | General | MEDLINE, Current Contents, and PsycINFO | 1966–2000 |
| McFadden 2011 [77] | Narrative review | St John’s wort, ginkgo, liquorice, garlic | General | No mention | No mention |
| Mills 2005 [78] | Systematic review | Natural health products | General | AMED, CINAHL, E-Psyche , CISCOM (2000–December 2002), Cochrane Central Register of Controlled Trials , and MEDLINE | Their inception to 2004 |
| Norred 2001 [79] | Narrative review | Herbs and dietary supplements with anticoagulant effects | General | No mention | No mention |
| Nutescu 2011 [80] | Narrative review | Dietary supplements | Warfarin | No mention | No mention |
| Nutescu 2006 [81] | Narrative review | Herbs and dietary supplements | Warfarin | No mention | No mention |
| Ohnishi 2004 [82] | Narrative review | Dietary supplements | General | No mention | No mention |
| Pal 2006 [83] | Narrative review | Herbs | General | No mention | No mention |
| Poppenga 2002 [84] | Narrative review | Herbs | General | No mention | No mention |
| Samuels 2005 [85] | Narrative review | Herbs | Anticoagulants | No mention | No mention |
| Singh YN 2005 [86] | Narrative review | Kava, St. John’s wort | General | No mention | No mention |
| Skalli S 2007 [87] | Systematic review | Common herbal, e.g., St. John’s wort, Garlic, Ginseng, Danshen, Ginkgo | General | MEDLINE via PubMed, Allied and Complementary, Medicine Database, HealthSTAR, AMBASE, CINHAL, Cochrane Library | 1966 to 2006 |
| Tachjian 2010 [88] | Narrative review | Herbs | Cardiovascular drugs | PubMed, MEDLINE | 1966-2008 |
| Tirona 2006 [89] | Narrative review | St John’s wort | General | No mention | No mention |
| Tomlinson 2008 [90] | Narrative review | Herbs | CYP3A4/p-gp substrates | No mention | No mention |
| Ueng 2004 [91] | Narrative review | Herbs involving CYP enzymes | General | No mention | No mention |
| Venkataramanan 2006 [92] | Narrative review | St. John's wort and Milk Thistle | General | No mention | No mention |
| Whitten 2006 [93] | Systematic review | St John’s wort | CYP3A substrates | MEDLINE, Cinahl, PsycINFO, AMED, Current Contents and EMBASE | Their inception to 2005 |
| Williamson 2003 [94] | Narrative review | Herbs | General | EMBASE, MEDLINE | Embase: 1980-2003; Medline: 1966-2003 |
| Williamson 2005 [95] | Narrative review | Herbs | Conventional medicines | No mention | No mention |
| Wittkowsky 2008 [96] | Narrative review | Herbs and dietary supplements | Anticoagulants | No mention | No mention |
| Woodward 2005 [97] | Narrative review | Homeopathic and herbal medicines | General | No mention | Their inception to 2005 |
| Yang 2006 [98] | Systematic review | Herbs | General | MEDLINE (via PubMed), Biological Abstracts, Cochrane Library, and EMBASE | Their inception to 2005 |
| Zhou 2005 [99] | Narrative review | Danshen | General | MEDLINE, SCIFINDER | 1982 to 2005 |
| Zhou 2004 [100] | Systematic review | St John's wort | General | MEDLINE (via PubMed), Biological Abstracts, PsycINFO, Cochrane Library, AMED (Allied and Complementary Medicine), Biosis Previews and EMBASE | Their inception to 2003 |
| Zhou 2003 [101] | Narrative review | Herbs involving CYP enzymes | Substrates of CYP enzymes | No mention | No mention |
| Zhou 2007 [102] | Systematic review | Herbs | General | MEDLINE (via PubMed), Biological Abstracts, Cochrane Library, and EMBASE | Their inception to 2007 |
| Mao 2007 [103] | Narrative review | Chinese herbal medicine | CYP3A4/p-gp substrates | No mention | No mention |
| Wang 2011 [104] | Narrative review | Dietary supplements | General | No mention | No mention |
| Wang 2003 [105] | Narrative review | Herbs | Warfarin/antiplatelets | No mention | No mention |
| Li 2008 [106] | Narrative review | Chinese herbal medicine | Warfarin | No mention | No mention |
| Lin 2003 [107] | Narrative review | Ginseng and Ginkgo biloba | General | No mention | No mention |
| Chen 2011 [108] | Narrative review | Traditional Chinese medicine | General | No mention | No mention |
| Chen 2007 [109] | Narrative review | Chinese herbal medicine | General | No mention | No mention |
| Peng 2011 [111] | Narrative review | Traditional Chinese medicine | General | No mention | No mention |
| Tsai 2009 [112] | Narrative review | Ginseng | Warfarin | MEDLINE (OVID), CJFT database | 2000 to 2008 |
| Lai 2007 [113] | Narrative review | Chinese herbal medicine | General | No mention | No mention |

CAM: complementary and alternative medicine; CYP: cytochrome P450; CJFT: China Journals Full-text.
